# Dentin Sialoprotein is a Novel Substrate of Matrix Metalloproteinase 9 *in vitro* and *in vivo*

**DOI:** 10.1038/srep42449

**Published:** 2017-02-14

**Authors:** Guohua Yuan, Lei Chen, Junsheng Feng, Guobin Yang, Qingwen Ni, Xiaoping Xu, Chunyan Wan, Merry Lindsey, Kevin J. Donly, Mary MacDougall, Zhi Chen, Shuo Chen

**Affiliations:** 1 The State Key Laboratory Breeding Base of Basic Science of Stomatology & Key Laboratory for Oral Biomedicine of Ministry of Education, School and Hospital of Stomatology, Wuhan University, Wuhan, 430079, China; 2 Department of Developmental Dentistry, University of Texas Health Science Center, San Antonio, 78229, USA; 3 Department of Surgery, The First Affiliated Hospital, Fujian Medial University, Fuzhou, 350005, China; 4 Department of Anatomy and Histoembryology, Fujian Medical University, Fuzhou, 350018, China; 5 Department of Engineering, Mathematics and Physics, Texas A&M International University, Laredo, 78041, USA; 6 Department of Periodontics, University of Texas Health Science Center, San Antonio, 78229, USA; 7 Department of Physiology and Biophysics, University of Mississippi Medical Center, Jackson, 39216, USA; 8 Department of Oral/Maxillofacial Surgery/Institute of Oral Health Research, University of Alabama at Birmingham School of Dentistry, Birmingham, 35294, USA

## Abstract

Dentin sialoprotein (DSP) is essential for dentinogenesis and processed into fragments in the odontoblast-like cells and the tooth compartments. Matrix metalloproteinase 9 (MMP9) is expressed in teeth from early embryonic to adult stage. Although MMP9 has been reported to be involved in some physiological and pathological conditions through processing substrates, its role in tooth development and whether DSP is a substrate of MMP9 remain unknown. In this study, the function of MMP9 in the tooth development was examined by observation of *Mmp9* knockout (*Mmp9*−/−) mouse phenotype, and whether DSP is a substrate of MMP9 was explored by *in vitro* and *in vivo* experiments. The results showed that *Mmp9*−/− teeth displayed a phenotype similar to dentinogenesis imperfecta, including decreased dentin mineral density, abnormal dentin architecture, widened predentin and irregular predentin-dentin boundary. The distribution of MMP9 and DSP overlapped in the odontoblasts, the predentin, and the mineralized dentin, and MMP9 was able to specifically bind to DSP. MMP9 highly efficiently cleaved DSP into distinct fragments *in vitro*, and the deletion of *Mmp9* caused improper processing of DSP in natural teeth. Therefore, our findings demonstrate that MMP9 is important for tooth development and DSP is a novel target of MMP9 during dentinogenesis.

Dentin is a major mineralized component of teeth. Odontoblasts, differentiated from neural-crest derived mesenchymal cells^1^, synthesize and secrete un-mineralized extracellular matrix (ECM) termed predentin containing rich non-collagenous proteins (NCPs). Later, when apatite crystals are deposited, the predentin is transformed to the mineralized dentin. Under normal conditions, a rather uniform layer of predentin is maintained between the mineralized dentin and the odontoblast layer, indicating that the rates of secretion and maturation of the predentin is identical and controlled by accurate mechanisms. NCPs have been proposed to be critical for this process^2^.

Dentin sialoprotein (DSP) and dentin phosphoprotein (DPP) are the two most abundant NCPs in dentin, and encoded by a single gene, dentin sialophosphoprotein (Dspp)^3^,^4^. *Dspp* knockout (*Dspp*−/−) mouse displays a phenotype similar to the manifestations of human dentinogenesis imperfecta III (DGI-III)^5^. Transgenic expression of mouse *Dsp* in the *Dspp*−/− background leads to a partial rescue of the *Dspp*−/− tooth phenotype with restored predentin width, an absence of irregular unmineralized areas in dentin and less frequent pulp exposure^6^. Heterogeneous mutations in DSP coding domain in humans have been reported to cause DGI-II and dentin dysplasia II^7^. These studies demonstrate that DSP, the NH_2_-terminus protein from DSPP, is essential to dentinogenesis.

Only trace amount of full-length DSPP protein was detected in extracts from the pulp/odontoblasts and dentin. Key proteolytic cleavage site of mouse DSPP into DSP and DPP has been determined at the NH_2_-terminal peptide bond of Asp^452^. The cleavage of DSPP into DSP and DPP is as an activation step of DSPP function, for failure to make this cleavage results in dentin and periodontal developmental defects^8^,^9^,^10^. Interestingly, DSP has been recently found to be further processed into small fragments in odontoblast-like cells and dental organs^11^,^12^,^13^. Our previous study has reported that cleaved DSP fragments are localized in different compartments of teeth. DSP NH_2_-terminal fragment(s) are mainly distributed in the non-mineralized predentin and odontoblasts, but weakly in the mineralized dentin, whereas DSP COOH-terminal fragment(s) are mainly restricted to the mineralized dentin rather than the predentin and odontoblasts. The distinct distribution pattern of DSP NH_2_-terminal and COOH-terminal fragments in the odontoblasts, predentin and dentin suggest that they may play distinct functions during dentinogenesis^11^. Besides, DSP and DSP-derived peptides are able to activate intracellular signaling transductions through regulating gene expression and protein phosphorylation, and induce dental primary/stem cell differentiation^14^,^15^,^16^. The above evidence suggested that, similar to DSPP, the proteolytic processing of DSP might also be an activation step of DSP biological function. However, the protease(s) catalyzing DSP proteolysis remained unknown.

Matrix metalloproteinases (MMPs) are a family of zinc- and calcium-dependent proteolytic enzymes. The involvement of MMPs in a wide variety of physiological development and pathological diseases has been reported through remodeling and degrading their extracellular, membrane-bound or intracellular substrate repertoire^17^. Experiments to chemically inhibit MMPs indicated that MMPs (possibly MMP2, 3, 9 and/or 20) played a critical role in the onset of dentin mineralization^18^. MMP9 belongs to gelatin-binding MMP and is known to be involved in bone development. Ablation of *Mmp9* in mice causes delayed skeletal growth plate ossification and defective bone fracture repair, and mutations of *Mmp9* in humans result in the human disease metaphyseal anadysplasia^19^,^20^,^21^. Bone and dentin are both mineralized connective tissues and share many common characteristics including composition and mechanisms of formation^2^. Therefore, we hypothesized that MMP9 may play critical roles during dentin formation through remodeling its substrate(s) in teeth. MMP9 has an overlapping distribution profile with DSP in the odontoblasts, predentin and dentin matrix^11^,^22^, suggesting that MMP9 might involve in dentin formation and process DSP. In this study, we showed the function of MMP9 in dentin formation by observation of *Mmp9*−/− tooth phenotype and revealed the possible underlying mechanism by experiments of *in vitro* and *in vivo* cleavage of DSP by MMP9.

## Results

### *Mmp9*−/− mouse teeth show severe cusp wear and increased reactionary dentin (RD) formation

**Figure 1:**
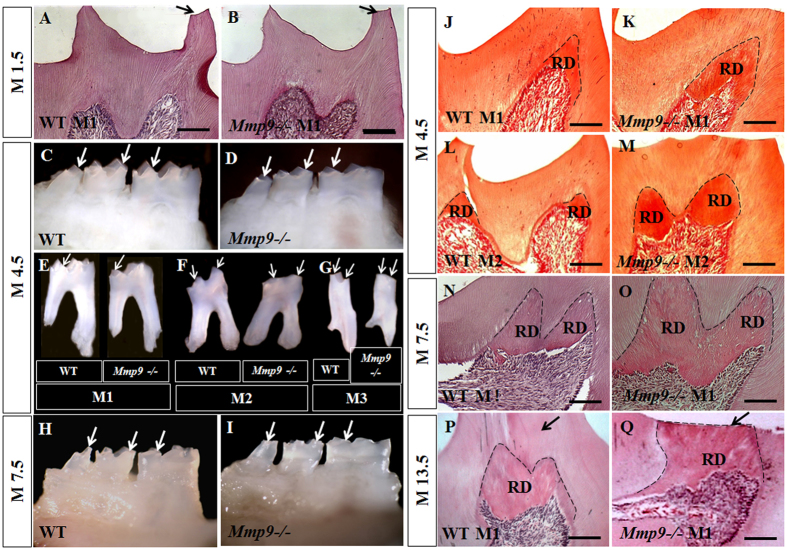
More severe molar cusp wear and increased reactionary dentin (RD) formation in the *Mmp9*−/− mice. (**A**,**B**) Slight wear of one cusp (arrows) and no RD formation was seen at M1.5 in both *Mmp9*−/− and wild-type molars. (**C**–**G**) At M4.5 and (**H**,**I**) M7.5, the heights of the cusps (arrows) in the *Mmp9*−/− mice were reduced compared with the controls. (**J**–**Q**) More amount of RD (dashed lines) was seen in the *Mmp9*−/− molars at M4.5, M7.5 and M13.5. At M13.5, dentin on top of RD (arrows) was worn off in the *Mmp9*−/− mice but not in the wild-type mice. WT, wild-type; M1, the first mandibular molar; M2, the second mandibular molar, M3, the third mandibular molar; RD, reactionary dentin. Scale bars: 100 μm. (**A**,**B**,**J**–**Q**) HE staining images; (**C**–**I**) Stereomicroscopy images.

To address the role of MMP9 for tooth development, we first examined the morphology of *Mmp9*−/− teeth from 1.5 months old (M1.5) to M13.5 by stereomicroscopy and histology. At M1.5, only a very slight wear of molar cusp was seen in the control and *Mmp9*−/− molars, and no RD was seen in either group (Fig. 1A,B). At M4.5 and M7.5, *Mmp9*−/− mice showed more severe wear of molar cusps (Fig. 1C–I). In response to severe wear, the amount of RD in *Mmp9*−/− molars was apparently increased compared to the control (Fig. 1J–O). At M13.5, the cusp wear progressed to the depth of RD in *Mmp9*−/− mice, but thick dentin tissue remained on the top of RD in the control mice (Fig. 1P,Q).

### Abnormal mineralization and structures of *Mmp9*−/− teeth

**Figure 2:**
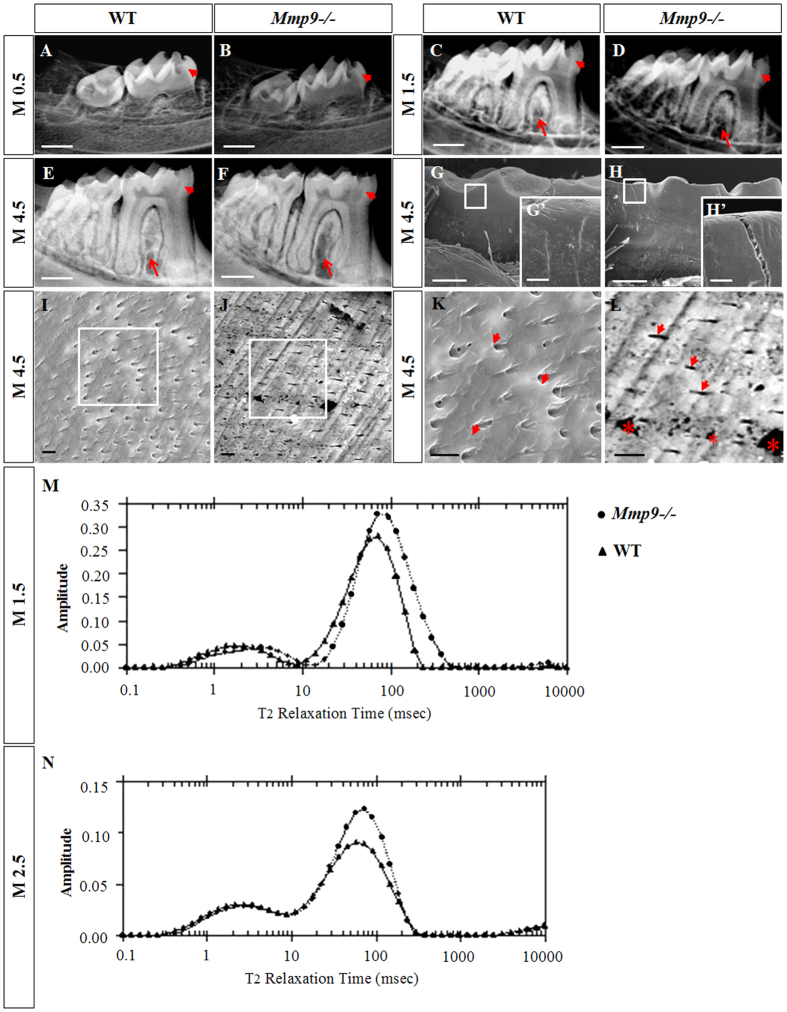
Decreased mineral density of teeth, loss of the alveolar bone in the furcation region, and abnormal tooth structures in the *Mmp9*−/− mice. (**A**–**F**) Representative radiographs show an overall reduction of the mineralization of the molars (arrowheads) and loss of the alveolar bone in the furcation region (arrows) of the *Mmp9*−/− mice. (**G**,G’,**H**,H’) SEM analysis showed the presence of abnormal fissure on the surface of the *Mmp9*−/− molars. (**I**–**L**) The dentin tubules (arrowheads) and the intertubular dentins were well-distributed in the wild-type mice, but in the *Mmp9*−/− mice the distribution of the dentine tubules (arrowheads) and the intertubular dentins were not uniform with decreased number of dentin tubules and numerous “holes” (*) in the intertubular dentins. K and L are higher magnifications of the rectangles in (**I** and **J**), respectively. (**M**,**N**) Inversion T2 relaxation time spectra for the first mandibular molars of the wild-type and the *Mmp9*−/− mice at M1.5 and M2.5. The lines with “dots” are for the *Mmp9*−/− molars and the lines with “triangles” are for the wild-type molars. Longer T2 relaxation time and larger area under the curve was found in the *Mmp9*−/− molars. The longer T2 relaxation time is corresponding to larger pores, and the larger area is corresponding to larger porosity (larger amount of holes). WT, wild-type. Scale bars: (**A**–**J**) 500 μm, (**K**,**L**) 250 μm, (K’,L’) 20 μm, (M-P) 5 μm.

To further understand why severe cusp wear occurred in *Mmp9*−/− molars, possible changes in the mineralization and structures of *Mmp9*−/− teeth were examined by radiography and SEM. Decreased mineral density of the dentin and the enamel in the *Mmp9*−/− molars was seen from M0.5 to M4.5 by radiography (Fig. 2A–F). SEM analysis showed that the molar enamel surfaces in the M4.5 *Mmp9*−/− mice had abnormal fissures (Fig. 2G,H,G’,H’). Meanwhile, the dentinal tubules and inter-tubular dentin were uniformly distributed in the wild-type mice, but sparsely scattered at an obviously impaired density in *Mmp9*−/− mice. Numerous abnormal “holes” were also observed in the *Mmp9*−/− dentin (Fig. 2I–L). Then, NMR proton spin-spin (T2) relaxation time measurement was applied to analyze the porosity and pore size of molars at M1.5 and M2.5^23^. Inversion NMR T2 relaxation time spectra showed that *Mmp9*−/− molars had larger porosity and pore size compared to the wild-type control (Fig. 2M,N). These results indicated that teeth in *Mmp9*−/− mice had not only reduced mineral content but also abnormal architecture.

In addition, examination of the mandibles by radiography also identified alveolar bone defects in *Mmp9*−/− mice (Fig. 2C–F). The mineral density in the furcation region of mandibular molars decreased in *Mmp9*−/− mice, which is most notable when comparing the 4.5-month (Fig. 2E,F) samples. The significant alveolar bone loss was confirmed by hematoxylin and eosin (HE)-stained tissue sections (Supplementary Fig. 1).

### Delayed differentiation of odontoblasts, widened predentin, and irregular mineralization front in *Mmp9*−/− mice

**Figure 3:**
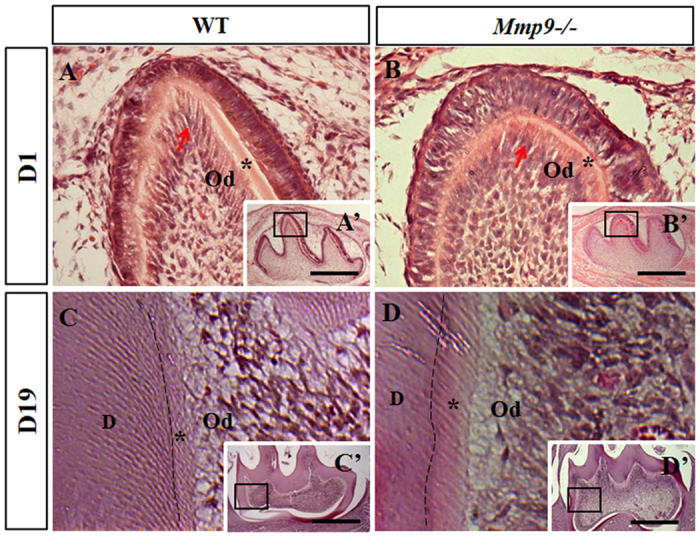
Delayed differentiation, widened predentin and irregular mineralization front in the *Mmp9*−/− molars. (**A**,A’,**B**,B’) At D1, the predentin matrix was deposited by well-differentiated odontoblasts (arrow) at the cusp tip of wild-type first mandibular molar. The polarization and elongation of odontoblasts (arrow) were delayed, and the thickness of predentin at the cusp area was reduced in the *Mmp9*−/− first mandibular molar cusp. (**C**,C’,**D**,D’) Compared to the control tooth, irregular mineralization front (dashed line) and enlarged predentin layer were observed in the *Mmp9*−/− molar at D19. (**A**–**D**) are higher magnifications of the rectangles in A’–D’, respectively. *predentin; D, mineralized dentin; Od, odontoblasts. Scale bars: (**A**–**F**) 50 μm, (**G**,**H**) 25 μm; (A’–H’) 500 μm.

To further observe the function of MMP9 during the odontoblast differentiation and secretion at earlier stages, *Mmp9*−/− teeth at D1 and D19 were analyzed using histological method. In wild-type mice at D1, the odontoblasts were well polarized and elongated at the cusp tip region, where the deposition of the predentin matrix was clearly discernible (Fig. 3A). In contrast, the polarization and elongation of odontoblasts were delayed, and the thickness of predentin at the cusp area was reduced in *Mmp9*−/− molars at D1 (Fig. 3B). At D19, increased predentin width and irregular predentin-dentin boundary was seen in *Mmp9*−/− molars versus the control (Fig. 3C,D).

### Overlapping expression of MMP9 and DSP in the odontoblast-like cells and the developing teeth

**Figure 4:**
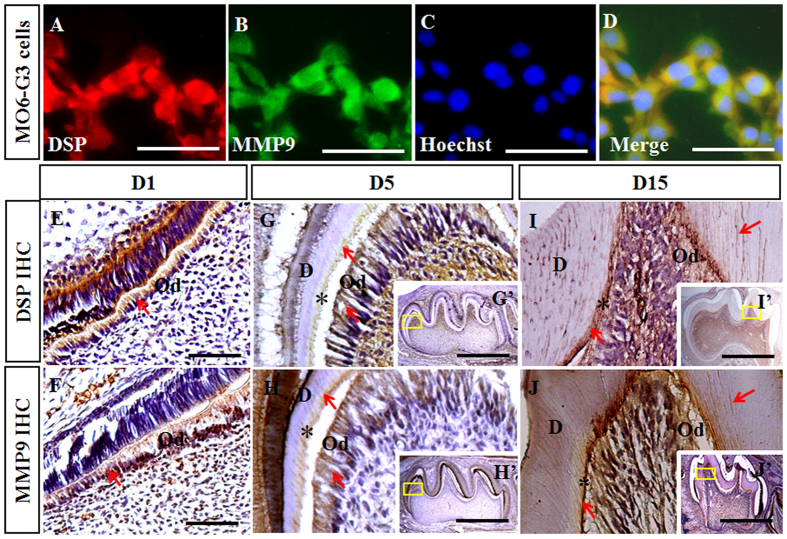
Overlapping distribution of DSP and MMP9 in the odontoblast-like cells and the developing teeth. (**A**,**B**) Immunofluorescent signals of DSP and MMP9 were observed in the cytoplasm of MO6-G3 cells. (**C**) Nuclei of the cells were counterstained with Hoechst. (**D**) Images of A-C were merged. (**E**,**F**) DSP and MMP9 were highly expressed in the odontoblasts at D1. (**G**-**J**, G’–J’) Immunoreactions of DSP and MMP9 were strong in the odontoblasts and the predentin, but weak in the mineralized dentin at D5 and D15. (**G**–**J**) are higher magnifications of the boxes in G’–J’, respectively. *predentin; Od, odontoblasts; D, the mineralized dentin. Scale bars: (**F**,**G**,**J**,**K**) 100 μm, (**H**,**I**,**L**,**M**) 50 μm, (H’,I’,L’,M’) 750 μm.

MMP9 and MMP2 belong to gelatinase subgroup of MMP family and share many common substrates^24^. DSP is critical for dentinogenesis and a substrate of MMP2 in the porcine molars^7^,^12^. Therefore, we hypothesized that DSP might act as a substrate of MMP9, which may explain the dentin phenotype of *Mmp9*−/− mice. To assess whether MMP9 is able to cleave DSP, we first confirmed co-distribution of DSP and MMP9 proteins in the mouse odontoblast-like (MO6-G3) cells and tooth tissues. Double immunofluorescence experiments showed that expression of MMP9 and DSP overlapped in the cytoplasm of MO6-G3 cells (Fig. 4A–D). Immunohistochemistry showed that in molars at D1, DSP and MMP9 were both highly expressed in the odontoblasts (Fig. 4E,F). At D5 and D15, signals of DSP and MMP9 were intense in the odontoblasts and the predentin, and mild in the mineralized dentin (Fig. 4G–J,G’–J’).

### Binding between DSP and MMP9

**Figure 5:**
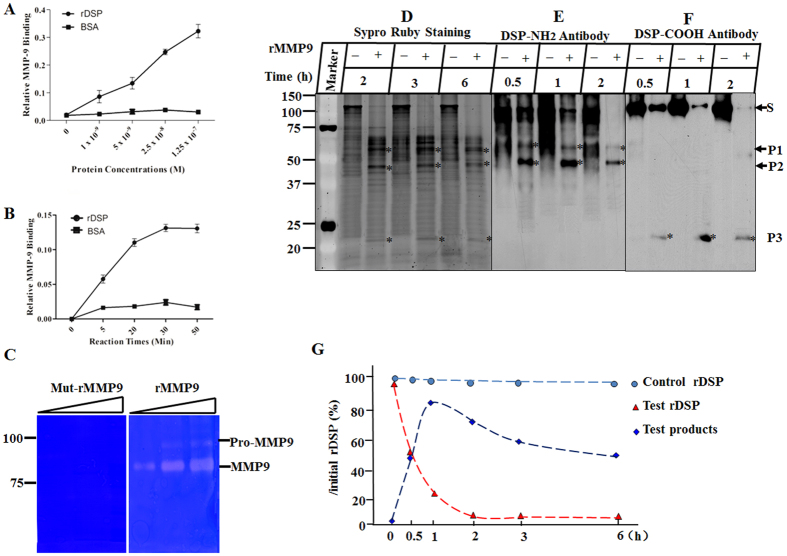
Recombinant DSP protein bound to MMP9 and was processed by MMP9 *in vitro*. (**A**,**B**) DSP bound to MMP9 in the dose- and time-dependent manner. (**C**) Gelatin zymography was performed to characterize the cleavage activity of rMMP9 and mut-rMMP9 with ascending concentrations of enzyme from the left the right lanes. Two clear bands at 92 kDa and 86 kDa were seen in the gel containing rMMP9, but not in the gel containing mut-rMMP9. (**D**) rDSP was incubated alone or with activated rMMP9 for 2, 3 and 6 h. The reaction products were run on SDS-PAGE gels and stained with Sypro Ruby dye. (**E**,**F**) Western blot assays were performed using anti-DSP-NH_2_ and -COOH antibodies, respectively. S, P1, P2, and P3 represent the substrate and cleaved products of rDSP. (**G**) rDSP and cleaved products were quantified when digested for 0, 0.5, 1, 2, 3 and 6 h with initial rDSP as 100%. Approximately 50% of the rDSP substrate was digested after 30 min of incubation, and almost all of the substrate was cleaved after 2 h. The quantity of the products reached the maximum at 1 h, and gradually reduced afterwards.

We next determined whether MMP9 was able to bind to DSP. First, rDSP fusion protein was generated, purified, and confirmed by Coomassie Blue staining and Western blot assays using anti-DSP and anti-GST antibodies as described previously^16^. Then the rDSP protein was labeled with biotin and serial dilutions of the biotinylated rDSP were incubated with either mut-rMMP9 or BSA. The results showed that rDSP bound specifically to MMP9 in the dose- and time-dependent manner whereas there was no binding effect on control BSA (Fig. 5A,B).

### DSP processed by MMP9 *in vitro*

To verify that rMMP9 was successfully activated and had the ability to cleave its substrates, gelatin zymography was first performed with rMMP9 and mut-rMMP9. Our data showed that rMMP9 efficiently digested gelatin with two clear bands at the molecular weight of 92 kDa and 86 kDa, whereas mut-rMMP9 failed to do so (Fig. 5C). Next, to determine whether the activated rMMP9 was able to process DSP protein, the rDSP was incubated alone or with rMMP9 for different time periods. The results showed that rDSP was cleaved by rMMP9 into three major fragments, whereas rDSP alone remained intact and stable (Fig. 5D–F). Western blot analyses demonstrated that the intact rDSP and the two cleaved fragments at high molecular weight (HMW) were recognized by anti-DSP-NH_2_ antibody (Fig. 5E), and the intact rDSP and the smallest cleaved rDSP product were detected by anti-DSP-COOH antibody (Fig. 5F). The cleaved products were quantified when digested for 0, 0.5, 1, 2, 3 and 6 h with initial rDSP substrate as 100%. The results showed that approximately 50% of the substrate was cleaved after 30 min of incubation, and the cleavage reaction was almost complete after 2 h. The products reached their maximum amount at 1 h, and were further processed by rMMP9 when incubated longer resulting in reduced remnant quantity (Fig. 5G).

**Figure 6:**
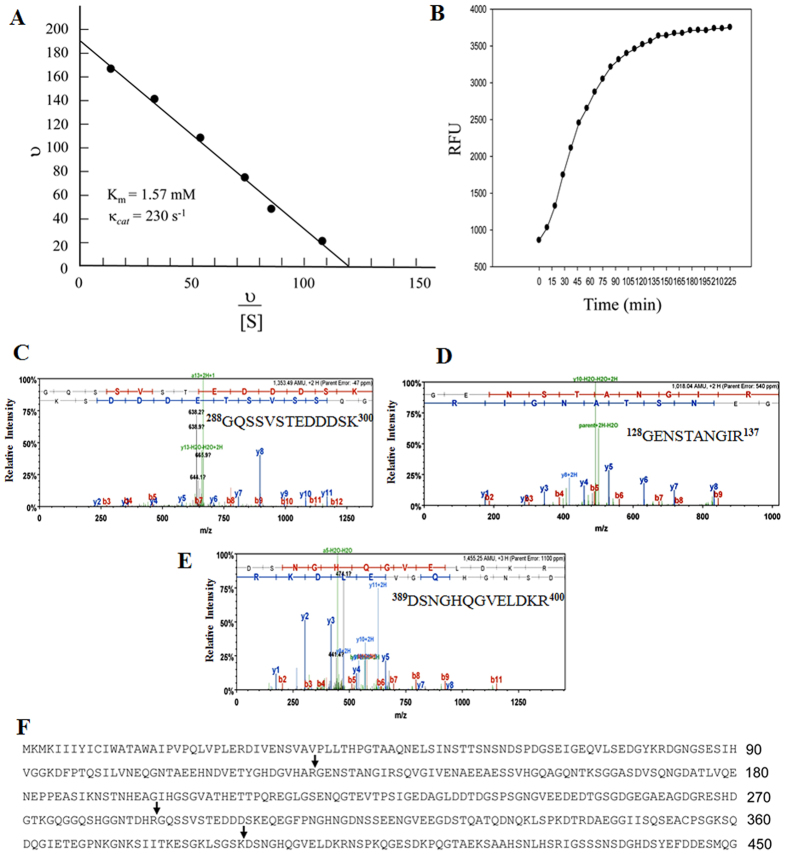
Enzyme kinetics and proteolytic sites of rDSP cleavage by MMP9. (**A**) An Eadie-Hofstee plot for MMP9 hydrolysis of rDSP. rDSP substrate ranging from 0.25 μM to 10 μM was incubated with the activated rMMP9 at a constant concentration (13.6 nM) in reaction buffer for 30 min at 37 °C. The reaction products were run on a SDS-PAGE gel and stained with Sypro Ruby dye. The density of the cleaved products was quantitated using imageJ software. (**B**) Activated rMMP9 was reacted with fluorescence-labeled MMP9 substrates (DNP-Pro-Cha-Gly-Cys(Me)-His- Ala-Lys(N-Me-Abz)-NH_2_). Gelatinase assays were carried out in 96-well microplates in assay buffer. Reactions were performed at 23 °C and the changes in fluorescence intensity were expressed in relative fluorescent units (RFU) as measured with λ_excitation_ = 365 nm and λ_emission_ = 450 nm in a SpectraMAX Gemini XS fluorescent plate reader. Data points represent an average of three separate experiments repeated in duplicate. (**C**–**F**) Mass spectrometry determined the sites of DSP cleavage by MMP9, including Arg127-Gly128 (P2), Arg287-Gly288 (P1), and Lys388-Asp389 (P3) peptide bonds.

To assess the catalytic efficiency of MMP9, steady state cleavage velocities were measured with a constant amount of rMMP9 and varying amounts of DSP substrate. As expected, the enzymatic reaction displayed reaction kinetics within a Michaelis-Menten analysis. An Eadie-Hofstee plot of these results showed Michaelis-Menten parameters were K_m_ = 1.57 mM and kcat = 230 (s^−1^), yielding a relative catalytic efficiency (kcat/K_m_) of 146, 500 M^−1^ s^−1^ (Fig. 6A). Similar to rDSP, MMP9 also had high efficiency in catalyzing a known fluorescent MMP9 substrate as a positive control (Fig. 6B)^25^. These results demonstrated that DSP was a novel substrate of MMP9.

To further identify the specific cleavage sites of DSP by MMP9, the three cleaved rDSP products were analyzed by mass spectrometry. The sites of DSP cleavage by MMP9 were determined as indicated in Fig. 6C–F. These results indicated that MMP9 selectively cleaved DSP protein.

### DSP processed by MMP9 *in vivo*

**Figure 7:**
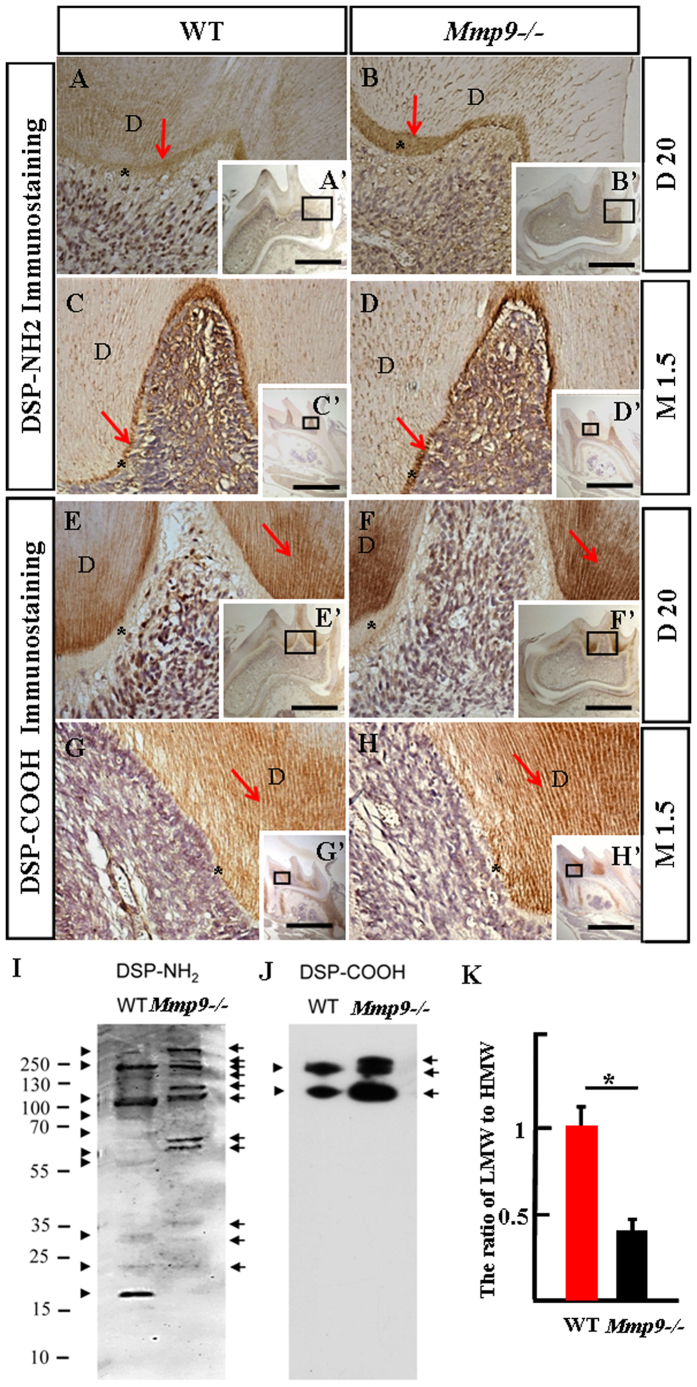
Immunolocalization and protein profiles of DSP in the *Mmp9*−/− and the wild-type molars. (**A**–**H**, A’–H’) Immunodistribution of anti-DSP-NH_2_ antibody was strong in the predentin and the odontoblasts, but weak in the mineralized dentin. In contrast, DSP-COOH fragment(s) was strong in the mineralized dentin, but weak in the predentin and the odontoblasts at D20 and M1.5. (**A**–**H**) are higher magnification of the boxes in A’–H’. (**I**,**J**) Proteins were isolated from the wild-type and the *Mmp9*−/− teeth at D15, and Western blots with anti-DSP-NH_2_ and –COOH antibodies were performed. For normalization, the protein concentration should be measured and same amount of protein was loaded. Different DSP protein profiles were seen in the wild-type and the *Mmp9*−/− teeth. Arrows and arrowheads show DSP bands from the *Mmp9*−/− and the wild-type teeth, respectively. (**K**) The densitometry of anti-DSP-NH_2_ and -COOH bands of three independent experiments was performed and relative quantification was processed with the ImageJ software. The ratio of the LMW fragments (lower than 95 kDa) to the HMW DSP (higher than 95 kDa) was calculated. Statistical analysis was performed using Student’s *t*-test. *P < 0.05. In (**A**–**I**), *predentin; D, dentin. Scale bars: (**A**–**H**) 50 μm, (A’–H’) 1 mm.

To analyze whether DSP was a substrate of MMP9 *in vivo*, immunohistochemistry with anti-DSP-NH_2_ and -COOH specific antibodies were performed in *Mmp9*−/− first mandibular molars at D20 and M1.5 with wild-type first mandibular molars in parallel as control. Our results showed that immunoreactions for anti-DSP-NH_2_ antibody were intense in the predentin matrix and odontoblasts, but mild in the mineralized dentin (Fig. 7A–D,A’–D’); for anti-DSP-COOH antibody, immunoreactions were apparently strong in the mineralized dentin, but weak in the predentin and odontoblasts (Fig. 7E–H,E’–H’). DSP signals recognized by both antibodies were more prominent in *Mmp9*−/− than control molars (Fig. 7A–H,A’–H’).

To further identify protein profiles of DSP fragments, proteins were isolated from wild-type and *Mmp9*−/− mouse teeth at D15 and Western blots were conducted using anti-DSP-NH_2_ and -COOH antibodies. The results showed different expression patterns of DSP in the wild-type and *Mmp9*−/− teeth. For anti-DSP-NH_2_ antibody, several high HMW bands (ranging from 95 to over 250 kDa) were seen in the *Mmp9*−/− but not in wild-type tooth proteins. Several low molecular weight (LMW) DSP bands (lower than 95 kDa) were detected in the wild-type and the *Mmp9*−/− teeth, but at a higher molecular weight in the *Mmp9*−/− teeth (Fig. 7I). For anti-DSP-COOH antibody, one additional HMW DSP band was observed in Mmp9−/− teeth but not in wild-type teeth (Fig. 7J). The ratio of the LMW fragments to the HMW DSP was significantly lower in the Mmp9−/− teeth compared to the control (Fig. 7K). Thus, MMP9 activity was responsible for DSP processing in natural dental tissues.

Osteopontin (OPN) has been reported to be localized in tooth tissues including predentin, dentin and RD^11^. And OPN is known as a substrate of MMP9^26^. Therefore, OPN expression changes in the *Mmp9*−/− mice from D17 to M7.5 were investigated by immunohistochemistry. OPN showed a higher expression level in the predentin, dentin and RD in the *Mmp9*−/− molars (Supplementary Fig. 2).

## Discussion

Heterogeneous mutations in DSP coding domain are associated with human hereditary dentin defects^7^. MMP9 and DSP are both highly expressed by the odontoblasts and secreted to the dentin ECM (Fig. 4)^11^,^22^. In this study, to gain insights of the role of MMP9 in the tooth development and whether DSP acts as a substrate of MMP9, we investigated the phenotype of *Mmp9*−/− teeth as well as the processing of DSP by MMP9 *in vitro* and *in vivo*. Our results showed that *Mmp9*−/− mice displayed phenotype similar to human DGI, including severe tooth wear, decreased dentin mineralization, delay of the odontoblast differentiation, and abnormal dentin structures. MMP9 was able to bind to and specifically process DSP into given fragments *in vitro. In vivo* DSP processing was affected in the *Mmp9*−/− teeth, which may mainly account for the dentin defects in the *Mmp9*−/− mice.

Previous studies have reported developmental abnormality of long bone in the mice deficient of *Mmp9* and in humans carrying *Mmp9* mutations^19^,^20^,^21^. Bone and dentin share many similarities in composition and mechanisms of formation^2^. Therefore, we hypothesized that dentin formation might be affected by deletion of *Mmp9*. In this study, we investigated the tooth phenotype of the *Mmp9*−/− mice by multiple methods including stereomicroscopy, histology, radiography, SEM and NMR. *Mmp9*−/− molars from M4.5 to M13.5 displayed more severe tooth cusp wear by stereomicroscopy and histology (Fig. 1), suggesting that the resistance to masticatory mechanical strength of *Mmp9*−/− molars diminished. SEM and radiography experiments did show abnormal appearance of dentin structure, and reduced dentin mineral density in the *Mmp9*−/− mice. In addition, the enamel in the *Mmp9*−/− molars was observed abnormal with fissures on the surface and showed reduced mineral density. Meanwhile, larger porosity and pore size were detected in the *Mmp9*−/− teeth by NMR (Fig. 2). Therefore, reduced mineral content of the enamel and abnormal tooth architecture may explain why *Mmp9*−/− teeth had lower mechanical resistance to masticatory forces. In this study, we focused on the dentin formation of the *Mmp9*−/− mice.

In response to severe tooth wear, more amount of RD was formed in *Mmp9*−/− molars from M4.5 to M13.5 compared to control molars at the corresponding ages (Fig. 1), suggesting that odontoblasts in *Mmp9*−/− molars are active in secreting dentin matrix to protect themselves from exposure to bacterial environment in oral cavity. This indicates that the capability of odontoblasts to synthesize and secret dentin ECM under masticatory force stimuli was not inhibited by deficiency of *Mmp9.*

The expression of MMP9 in the early embryonic dental epithelium and mesenchyme suggests a potential role of MMP9 in tooth morphogenesis^27^. However, *Mmp9*−/− molars and incisors did not show obvious morphological changes, indicating that MMP9 is dispensable for tooth morphogenesis. The delayed differentiation of odontoblasts in the *Mmp9*−/− molars (Fig. 3) is consistent with the expression of MMP9 in presecretory and secretory odontoblasts (Fig. 4). Later, MMP9 is present in the predentin and the dentin matrix (Fig. 4)^22^, suggesting that MMP9 may be involved in the dentin mineralization through processing and degrading dentin ECM, in accordance with which widened predentin and irregular boundary between predentin and dentin were detected in *Mmp9*−/− molars at D19 (Fig. 3).

The above experiments indicated the necessity of MMP9 activity for odontoblast differentiation and dentin formation, and showed that *Mmp9*−/− mice exhibited similar tooth phenotype to humans carrying *DSP* mutations^28^,^29^,^30^. Since DSP is processed into fragments in the odontoblast-like cells and the tooth compartment^11^,^13^, further studies were carried out to determine whether MMP9 participated in the DSP processing. Immunostaining experiments confirmed the overlapping distribution of MMP9 and DSP in the odontoblast-like MO6-G3 cells, and in the odontoblasts and dentin ECM of mouse molars (Fig. 4). DSP but not BSA bound to MMP9 in the dose- and time-dependent manner (Fig. 4). The overlapping localization and direct physical interaction between DSP and MMP9 are two prerequisites for processing of DSP by MMP9. Next, *in vitro* studies revealed that MMP9 selectively cleaved DSP protein into three major fragments (Fig. 5). Enzyme kinetics showed that MMP9 had a high efficiency for processing DSP with a turnover of (kcat/K_m_) of 146,000 M^−1^ s^−1^ (Fig. 6). This value is comparable to other substrates cleaved by MMP9^31^. For *in vivo* study, *Mmp9*−/− molars showed elevated expression levels of both DSP NH_2_-terminal and COOH-terminal fragments by immunohistochemistry (Fig. 7), suggesting that the processing of DSP was reduced in *Mmp9*−/− molars. Western blot assay was further performed and confirmed that expression patterns of DSP fragments were different between the control and the *Mmp9*−/− teeth. Several HMW DSP fragments were present in *Mmp9*−/− teeth but not in control molars and the ratio of the LMW DSP fragments to the HMW DSP was lower in the *Mmp9*−/− teeth (Fig. 7), assuring that DSP processing *in vivo* was affected by deletion of *Mmp9*. DSP is well known as a critical protein for proper dentin formation. Thus, the lack of appropriate DSP processing is likely the most important contributor to the dentin phenotype in *Mmp9*−/− teeth.

In addition to dentin defects, we revealed severe loss of alveolar bone surrounding *Mmp9*−/− molar roots, especially in the furcation region of the mandibular molars (Fig. 2 and Supplementary Fig. 1). The alveolar bone phenotype in *Mmp9*−/− mice is similar to *Dspp*−/− mice^10^. Like in dentin, DSPP is also proteolytically processed into DSP and DPP in bone. Processing of DSPP is essential to its biological function in alveolar bone, for disruption of DSPP processing into the given fragments failed to rescue the alveolar bone defect of *Dspp*−/− mice^9^,^10^. Therefore, in addition to dentin, the further cleavage of DSP by MMP9 might also exist in the alveolar bone, which is critical for alveolar bone remodeling. Further studies are needed to verify this possible underlying mechanism of alveolar bone loss in *Mmp9*−/− mice in the future.

OPN and DSP are both members of SIBLING (Small Integrin-Binding Ligand N-linked Glycoproteins) family. OPN shares many common characteristics with DSP^32^ and is known as a substrate of MMP9^26^. OPN has been reported in predentin, dentin and RD by our previous study^11^. As expected, improper processing and degradation of OPN in *Mmp9*−/− molars was documented by remarkably elevated protein level of OPN (Supplementary Fig. 2). Both *in vitro* and *in vivo* studies have shown that OPN is a potent inhibitor of hydroxyapatite crystal formation and growth^33^,^34^. However, the teeth of *Opn* deficient mice were found morphologically normal at the level of light and electron microscopy^35^. Therefore, compared to DSP, increased OPN protein might play a minor role for the dentin defects in *Mmp9*−/− molars. The decreased processing of OPN acts as a positive control for the improper processing of DSP in *Mmp9*−/− teeth.

Taken together, DSP is critical for tooth development, and DSP is a novel target of MMP9 during dentinogenesis. Like other MMPs, MMP9 was originally discovered to function in the breakdown of ECM. However, recent studies have identified intracellular proteins as MMP9 substrates^36^,^37^,^38^. The overlapping prominence of DSP and MMP9 in the cytoplasm of MO6-G3 cells and the existence of multiple LMW DSP fragments in MO6-G3 cell lysate suggest that the cleavage of DSP by MMP9 may be initiated inside odontoblasts^11^. Further studies are needed to determine the mechanisms of intracellular MMP9 activation and its cleavage of DSP to provide deeper insights into the processing of DSP by MMP9 during dentinogenesis.

## Methods

### Animal preparation

The protocol of animal use was approved by the Animal Welfare Committee at the University of Texas Health Science Center at San Antonio (UTHSCSA, Protocol No. 05012x). All experiments were performed in accordance with the relevant guidelines and regulations. The *Mmp9*−/− mice used in this study were originally generated and described by Vu and colleagues^19^. Genotyping was performed by PCR of genomic DNA extracted from tail snips (Supplementary Fig. 3). The tissue sections from the wild-type and *Mmp9*−/− mice were immunostained with anti-MMP9 antibody. MMP9 expression was seen in the wild-type tooth tissues, but not in *Mmp9*−/− mice (Supplementary Fig. 3). At least three mice for each time point were sacrificed and taken for the following analyses.

### Stereomicroscopy, X-radiography and low-field nuclear magnetic resonance (NMR)

To compare the overall morphology of wild-type and *Mmp9*−/− teeth, mice at the ages from M0.5 to M7.5 were put to death. Mandibles at M4.5 and M7.5 were observed under stereomicroscope. Mandibles at M4.5 were incubated in lysis buffer (2 × SSC, 0.2% SDS, 10 mM EDTA, 10 mg/ml proteinase K) for 2 d. After muscles surrounding teeth were digested, the molars were extracted and observed under stereomicroscope.

To compare the mineralization of teeth in *Mmp9*−/− mice with that in wild-type mice, mandibles from M0.5 to M4.5 were examined using a Faxitron radiograph inspection unit (Field X-ray Corporation, Lincolnshire, IL, USA).

To analyze the structural changes of *Mmp9*−/− teeth, the extracted first mandibular molars at M1.5 and M2.5 were processed for NMR measurement and compared with control as described previously^23^.

### Histology and immunohistochemistry

To evaluate the histological features of *Mmp9*−/− teeth, fresh mandibles at ages from D1 to M13.5 were fixed in 4% paraformaldehyde for 1–2 d at 4 °C, and demineralized in 8% EDTA for 1 d to 8 wk depending on age. Then, the tissues were processed for paraffin embedding and 5-μm serial sections were prepared and stained with HE.

To detect the distribution of MMP9, DSP, and OPN in mouse mandibular first molars, immunohistochemistry was performed as previously described^11^. Primary antibodies included goat anti-MMP9 antibody (Santa Cruz Biotechnology, CA, USA), two rabbit anti-DSP antibodies specific to the NH_2_ and COOH domains described previously^11^, and goat anti-OPN antibody (R & D systems, MN, USA). Normal goat or rabbit immunoglobulin G (IgG) (Santa Cruz Biotechnology, CA, USA) was employed as negative control (data not shown).

For double immunofluorescence, mouse odontoblast-like (MO6-G3) cells were grown on glass slides, rinsed with ice-cold phosphate-buffered saline (PBS), and fixed for 10 min on ice with methanol/acetone (1:1). Then, the cells were processed with blocking buffer for 1 h at room temperature (RT), and two primary antibodies were incubated simultaneously overnight at 4 °C (1:200 for rabbit anti-DSP antibody (M-300), 1:50 for goat anti-MMP9 antibody). After washes, slides were incubated with the secondary antibodies conjugated with Alexa Fluo ^®^ 486 green and Alexa Fluo ^®^ 568 red (1:500; Molecular Probes, OR, USA) for 1 h at RT. For nuclear staining, the cells were treated with Hoechst (Sigma-Aldrich, MO, USA).

### Scanning electron microscopy (SEM)

Mandibular incisors from *Mmp9*−/− and wild-type mice were fractured at the level of the labial alveolar crest. The first mandibular molars and the fractured incisor surfaces were fixed in 2% paraformaldehyde and 2.5% glutaraldehyde in 0.1 M cacodylate buffer. After washes, the samples were dehydrated in an ascending alcohol series, allowed to air dry, and then sputter-coated with gold for conventional SEM (JEOL, JSM 6610 LV; JEOL, Inc., Peabody, Mass., USA).

### Extraction of proteins from mouse teeth and detection of DSP

Molars from wild-type and *Mmp9*−/− mice were extracted as described above and tooth proteins were isolated by procedures described previously^6^. Extracted molars were crushed and incubated with 4 M guanidine HCl solution containing protease inhibitor (Sigma-Aldrich, MO, USA) overnight. The residue was then demineralized with 4 M guanidine HCl, 0.5 M EDTA plus protease inhibitors for 2 d. The solution was dialyzed against 4 M guanidine for 1 d, and against distilled water for another 2 d. Dialyzed solution was lyophilized and dissolved into distilled water. Protein concentrations were then measured.

Same amount of protein from wild-type and Mmp9−/− molars was loaded and subjected to Western blot assay.

### Expression and purification of recombinant DSP and MMP9

The cDNA coding for recombinant DSP (rDSP) was subcloned into pGEX-6P1 vector tagged with a NH_2_-terminal GST (GE Healthcare Biosciences, NJ, USA), termed GST-rDSP. To obtain mutant recombinant MMP9 protein (mut-rMMP9) with ligand-binding property but without catalytic activity, point mutation of the active site Glu_402_ of proMMP9 to alanine residue was generated as we previously described^39^. The constructs of GST-rDSP and mut-rMMP9 were transformed to E. coli BL21 (DE3). GST-rDSP and mut-rMMP9 proteins were induced and purified^16^. A wild-type recombinant mouse MMP9 protein (rMMP9) was purchased (R & D Systems, MN, USA).

### Gelatin zymography

To analyze their gelatinolytic activities, rMMP9 and mut-rMMP9 proteins were run on SDS-polyacrylamide gel electrophoresis (SDS-PAGE) containing 0.1% gelatin. The gelatin zymography gel was next washed in 2.5% Triton X-100 with gentle agitation for 1 h at RT, then incubated in the developing buffer (30 mM Tris-HCl, 200 mM NaCl, 5 mM CaCl_2_ and 1 mM ZnCl_2_) at 37 °C overnight. Counterstaining with Coomassie Brilliant Blue revealed gelatin degradation as clear bands corresponding to the enzymes against a blue background.

### Biotinylation of rDSP and substrate binding assay

For probing protein–protein interactions, rDSP protein at the concentration of 300 μg/ml was dialyzed against 0.1 M NaHCO_3_ and then reacted with 100 μg/ml Sulfo-NHS (N-hydroxysuccinimido)-LC (long-chain)-biotin (Pierce, Rockford, IL, USA) for 20 min at RT, followed by 2 h at 4 °C. Free biotin was removed by dialysis against 50 mM Tris-HCl and 150 mM NaCl, pH 7.4. To characterize the relative rDSP interaction with MMP9, substrate binding assay was performed as described earlier^40^. Briefly, 96-microwell plates were coated with 1 μg/well of mut-rMMP9 or bovine serum albumin (BSA) as control overnight at 4 °C and non-specific binding were blocked with 1% BSA (Sigma-Aldrich, MO, USA) for 1 h at RT. After thorough rinses with PBS, the biotinylated rDSP ranged from 0–18 fold molar excesses in PBS with 1% BSA was added into the plates and incubated for 30 min. Bound rDSP was reacted with AP-conjugated streptavidin diluted 1:10,000 in PBS for different time points using 1 mg/ml PNPP (ρ-nitrophenyl phosphate disodium) as substrate (Pierce, Rockford, IL, USA) and quantified at 405 nm (Opsys MR, Dynex, Chantilly, VA, USA). BSA was used as control group and the binding of rDSP to mut-rMMP9 was expressed as relative binding activity compared to the control group. The experiments were performed three times in triplicate.

### Processing of rDSP by rMMP9 *in vitro*

To quantify MMP9 activity, rMMP9 and mut-rMMP9 were reacted with fluorescence-labeled MMP9 substrate {DNP-Pro-Cha-Gly-Cys(Me)-His-Ala-Lys(N-Me-Abz)-NH_2_} (Calbiochem, Cambridge, MA, USA) as described previously^25^. In brief, gelatinase assays were carried out in 96-well microplates in total reaction volumes of 200 μl with assay buffer (50 mM Tris/HCl, 200 mM NaCl, 5 mM CaCl_2_, 20 mM ZnSO_4_, 0.05 Brij 35, pH 7.6). Reactions were performed at 23 °C and the changes in fluorescence intensity were expressed in relative fluorescent units (RFU) as measured with λ_excitation_ = 365 nm and λ_emission_ = 450 nm in a SpectraMAX Gemini XS fluorescent plate reader (Molecular Devices, Sunnyvale, CA, USA).

To assay the role of rMMP9 on rDSP processing, rDSP was incubated alone or in combination with P-aminophenylmercuric acetate (APMA)-activated rMMP9 (enzyme : substrate ratio of 1:20) in reaction buffer (50 mM Tris/HCl, 200 mM NaCl, 5 mM CaCl_2_, 20 mM ZnSO_4_, 0.05% Brij 35, pH 7.6). The reactions were carried out at 37 °C for various time intervals and stopped by adding 2 x SDS-PAGE sample buffer containing 2-mercaptoethanol. Samples were boiled for 5 min and subjected to SDS-PAGE and stained with Sypro Ruby dye. To detect DSP fragments in the digestion mixture more specifically, Western blot assay was performed using anti-DSP-NH_2_ and -COOH antibodies.

### Analysis of enzyme kinetics

Kinetics constants Km and kcat were determined from an Eadie-Hofstee plot of initial velocities under multiple turnover conditions^31^. Mixtures of the rMMP9 (13.6 nM) and substrate rDSP (0.25, 0.5, 1.0, 2.5, 5.0, 10 μM) were incubated in reaction buffer for 30 min at 37 °C. The reaction products were run on SDS-PAGE gel, stained with silver dye, and quantified with ImageJ. All experiments were performed in triplicate.

### Mass spectrometry

Twelve μg of purified rDSP were incubated with 0.6 μg of APMA-activated rMMP9 at 37 °C for 1 h in reaction buffer. Products were run on SDS-PAGE gel and visualized by Sypro Ruby dye staining. Then the products were excised from the gel, digested with trypsin, and the peptide mixtures were extracted from the gel as described by Kerkhoff *et al*.^41^. MALDI-mass spectrometry was performed using a TofSpec-2E instrument (Micromass, Manchester, UK) provided by Protein Core Facility of UTHSCSA.

### Statistical analysis

Quantitative data were presented as means + S. D. from three independent experiments and analyzed with Student’s *t* test. The differences between groups were statistically significant when P < 0.05. For the Western blot, densitometry of immunoblot bands was collected and relative quantification was processed with the ImageJ.

## Additional Information

**How to cite this article**: Yuan, G. *et al*. Dentin Sialoprotein is a Novel Substrate of Matrix Metalloproteinase 9 *in vitro* and *in vivo. Sci. Rep.* **7**, 42449; doi: 10.1038/srep42449 (2017).

**Publisher's note:** Springer Nature remains neutral with regard to jurisdictional claims in published maps and institutional affiliations.

## Supplementary Material

Supplementary Information
