# Supplementary material for: Dentin Sialoprotein is a Novel Substrate of Matrix Metalloproteinase 9 in vitro and in vivo
Source: Sci Rep. 2017 Feb 14;7:42449. doi: 10.1038/srep42449 (PMC5307955; doi:10.1038/srep42449)
Supplement: Supplementary Information [file srep42449-s1.pdf]

Dentin Sialoprotein is a Novel Substrate of Matrix Metalloproteinase 9 in vitro and in vivo

Guohua Yuan[1,2], Lei Chen[2,3], Junsheng Feng[2,4], Guobin Yang[1,2], Qingwen Ni[5], Xiaoping Xu[6], Chunyan Wan[2], Merry Lindsey[7], Kevin J. Donly[2], Mary MacDougall[8], Zhi Chen[1*], Shuo Chen[2*]

[1]The State Key Laboratory Breeding Base of Basic Science of Stomatology & Key Laboratory for Oral Biomedicine of Ministry of Education, School and Hospital of Stomatology, Wuhan University, Wuhan, 430079, China
[2]Department of Developmental Dentistry, University of Texas Health Science Center, San Antonio, 78229, USA
[3]Department of Surgery, The First Affiliated Hospital, Fujian Medial University, Fuzhou, 350005, China
[4]Department of Anatomy and Histoembryology, Fujian Medical University, Fuzhou, 350018, China
[5]Department of Engineering, Mathematics and Physics, Texas A&M International University, Laredo, 78041, USA
[6]Department of Periodontics, University of Texas Health Science Center, San Antonio, 78229, USA
[7]Department of Physiology and Biophysics, University of Mississippi Medical Center, Jackson, 39216, USA
[8]Department of Oral/Maxillofacial Surgery/Institute of Oral Health Research, University of Alabama at Birmingham School of Dentistry, Birmingham, 35294, USA

Supplementary Material

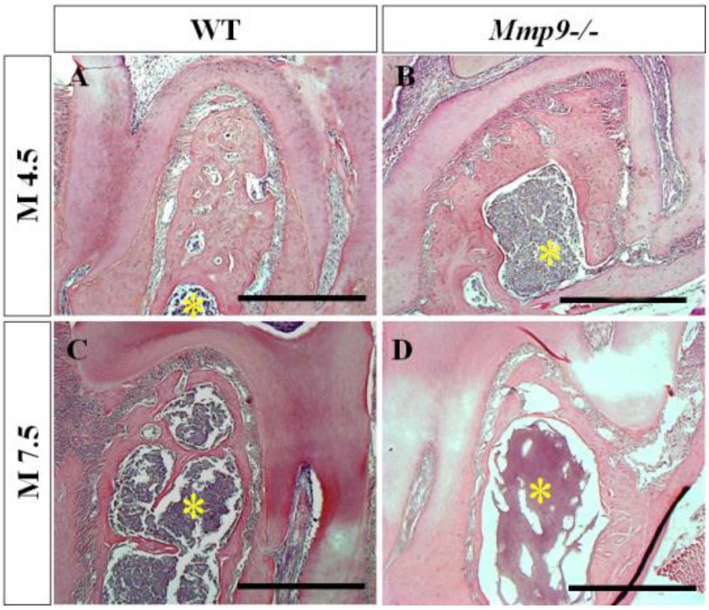

Supplementary Figure 1. Loss of mandibular alveolar bone in the furcation region of the *Mmp9-/-* mice. (A-D) HE staining demonstrated that the alveolar bones (*) in the furcation region of the first mandibular molars were resorbed in the *Mmp9-/-* mice compared with the wild-type mice. Scale bars: 500 µm.

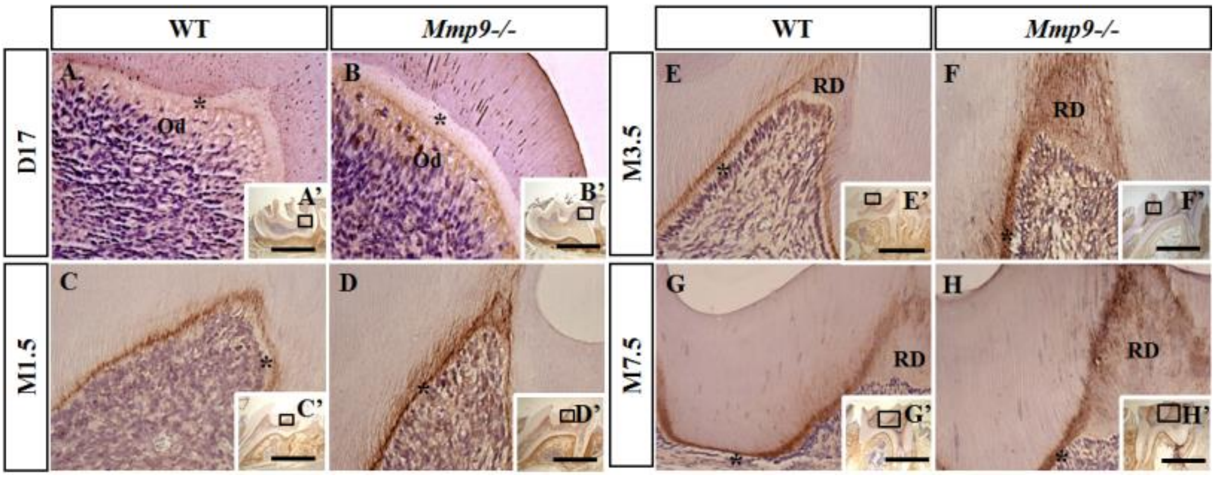

Supplementary Figure 2. Expression changes of OPN in *Mmp9-/-* molars. (A, A', B, B') At D17, OPN protein showed moderate expression at the mineralization front (arrow) and weak expression in the odontoblasts and the predentin in the wild-type molar, whereas in the *Mmp9-/-* molar, OPN immunoreactions were enhanced in the odontoblasts and downregulated in the mineralization front. (C, C', D, D') At M1.5, OPN antibody strongly stained the mineralization front and barely stained the predentin in the wild-type molar. In contrast, OPN was highly expressed in the predentin layer of the *Mmp9-/-* molar. (E-H, E'-H') At M3.5 and M7.5, anti-OPN antibody showed strong reactions in both the mineralization front and the RD with a higher expression level in the *Mmp9-/-* molars. Intense immunoreactions in the predentin layer were only seen in the *Mmp9-/-* molars. A-F are higher magnifications of the rectangles in A'-F', respectively. *P<0.05, **P<0.01. *predentin; Od, odontoblasts; RD, reactionary dentin. Scale bars: (A-H) 50 μm, (A'-H') 1 mm.

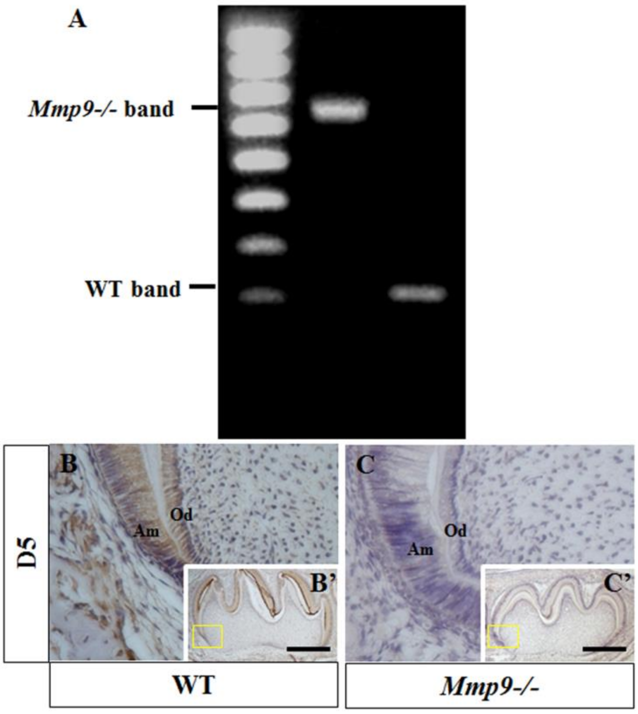

Supplementary Figure 3. Identification of the wild-type and *Mmp9-/-* mice. (A) Genotyping by PCR. Tail genomic DNA was isolated from the wild-type and the *Mmp9-/-* mice. Wild-type forward primer: 5'-GCATACTTGTACCGCTATGG-3'; reverse primer: 5'-TAACCGGAGGTGCAAACTGG-3'. *Mmp9-/-* forward primer: 5'-CTCAGAAGAACTCGTCAAGA-3'; reverse primer: 5'-GGATTGCACGCAGGTTCTCC-3'. The PCR products were run on 1% agarose gel and stained with ethidium bromide. The PCR band is 300 bp for wild-type mice, and 750 bp for *Mmp9-/-* mice. (B, B', C, C') Immuno-detection of MMP9 protein with anti-MMP9 antibody on the tissue sections of postnatal day 5 (D5) mice showed strong expression of MMP9 protein in the odontoblasts, ameloblasts and surrounding tissues in the wild-type mouse teeth, but no MMP9 staining was observed in the *Mmp9-/-* tissues. B and C show high magnification of the boxes in B' and C'. Scale bars: (B, C) 500 µm, (B', C') 50 µm.
